# Response to a report and press release by Bauer-Panskus and Then (2014) criticizing the presentation and interpretation of the results of recently published 90-day feeding studies with diets containing genetically modified MON810-maize varieties and their comparators (Zeljenková et al. 2014)

**DOI:** 10.1007/s00204-014-1429-x

**Published:** 2014-12-04

**Authors:** Pablo Steinberg

**Affiliations:** Institute for Food Toxicology and Analytical Chemistry, University of Veterinary Medicine Hannover, Bischofsholer Damm 15, 30173 Hannover, Germany

Dear Editors,

This letter is sent in response to a report and press release dated 7 November 2014 by Bauer-Panskus and Then (2014) which was issued on the website of TESTBIOTECH e.V. 7 November 2014. The authors criticize the presentation and interpretation of the results of recently published 90-day feeding studies with diets containing genetically modified MON810-maize varieties and their comparators (Zeljenková et al. 2014), which was performed within the EU-funded GRACE project. In the following paragraphs, we reply point-by-point to the criticisms raised:

1. Bauer-Panskus and Then claim: “The GLP-controlled 90-day feeding study in Han Wistar RCC rats aimed at providing a safety evaluation of two varieties of MON810 as a dietary admixture of 11 % (low dose) or 33 % (high dose). The authors claim to have followed “the guidance for such studies published by the EFSA Scientific Committee in 2011 and the OECD Test Guideline 408”. Although this is generally true, it needs to be stated that it is not entirely true, because the histopathological assessment of macroscopic findings (in the low dose group) as required in Test Guideline 408 was obviously not followed”.

*Response* In contrast to the statement of Bauer-Panskus and Then (2014), each gross lesion, as required by the OECD Test Guideline 408, was microscopically analysed. The macroscopic as well as the corresponding histopathological examination findings are shown in the Electronic Supplementary Material Table 6 of the publication by Zeljenková et al. (2014).

2. Testbiotech states that Zeljenková et al. (2014) “dismiss the toxicological relevance of the observed lower levels of total protein in serum (TP)”.

*Response* The TP level was significantly lower in the serum of male rats fed the 11 % GMO and 33 % GMO diet in the feeding trial A and in that of female rats fed the 33 % GMO diet in the feeding trial B if compared to the corresponding control animals. When assessing the biological/toxicological relevance of these above-mentioned results, one must take into account:

I. No such decreases were observed in the female rats fed the 11 % GMO and 33 % GMO diets in the feeding trial A, as well as in the male rats fed the 11 % GMO and 33 % GMO diet in the feeding trial B. The changes in TP are therefore inconsistent across the various groups and treatments. It should be noted that in both trials A and B genetically modified maize with the same GMO event (MON810) had been used. Moreover, a significantly lower total protein level in serum was also observed in female rats of study B fed the conventional maize DKC6815 at a 33 % level.
II. If the decrease of the serum protein levels were as relevant, as Bauer-Panskus and Then (2014) state, one would have expected that this decrease had an impact on the growth of the young animals used in these feeding trials. However, there were no significant body weight differences between rats fed the GMO diets and rats fed the control diets.
III. Bauer-Panskus and Then (2014) argue that the decrease in the serum total protein levels could be due to a nephrotic syndrome or to an impaired protein synthesis in the liver. If the rats were suffering from a nephrotic syndrome, oedema and histological alterations of the kidneys would be evident. In the experimental groups showing a decrease in serum total protein levels, neither oedema nor histological alterations of the kidneys were observed in male rats fed the 33 % GMO diet in feeding trial A and in female rats fed the 33 % GMO diet in feeding trial B. Furthermore, if protein synthesis were impaired, a certain degree of liver parenchymal cell death would have become evident, but the histopathological analyses of the liver of male rats fed the 33 % GMO diet in feeding trial A and in female rats fed the 33 % GMO diet in feeding trial B revealed no histological alterations.

Based on the above-mentioned facts, we conclude that the changes in TP in serum of male rats fed the 11 % GMO and 33 % GMO diet in the feeding trial A and in serum of female rats fed the 33 % GMO diet in the feeding trial B are not related to the feeding of the GMO-containing diets.

3. Bauer-Panskus and Then (2014) state “The second inappropriate dismissal involves weight changes of the pancreas and blood glucose levels. It is remarkable that the authors did not discuss these changes on a physiological level, in spite of the well-known role of the pancreas in the regulation of blood glucose levels”.

*Response* When wanting to analyse the biological/toxicological relevance of the pancreas weight and blood glucose level changes, one should take into account the following facts:

I. The statistically significant decrease in the relative pancreas weight of male rats fed the 11 % and 33 % GMO diet compared with the control group in the feeding trial A was not dose dependent. Lower pancreas weights were also observed in male rats fed the two conventional maize varieties in the feeding trial A; in the case of the conventional variety 2, the mean value was close to those of the two groups fed the GM diets. The slightly and not significantly decreased pancreas weights of male rats in feeding trial B also showed no correlation with the dose, and the mean value for the high dose group was practically identical with that of one of the groups fed conventional maize. Furthermore, in both trials, there were no significant differences in the pancreas weights of female rats.
II. The significant increase in the blood glucose levels of male rats fed the 11 % and 33 % GMO diet in the feeding trial A was not dose dependent. The measured blood glucose values were close to those of male rats fed the conventional maize varieties. No significant differences in blood glucose levels were observed between female rats fed the GMO diets in the feeding trial A and the corresponding control animals as well as between male and female rats fed the GMO diets in the feeding trial B and the corresponding control animals.
III. Considering the potential link between lower pancreas weights and elevated glucose levels in male animals, as claimed by Bauer-Panskus and Then (2014), it should be noted that serum glucose levels are influenced by a number of factors. Higher levels may, for example, result from feed consumption before sampling or stress. In the absence of changes in other relevant parameters, they should not be regarded as indicative of specific organ toxicity.
IV. In both trials, animals fed the GMO-containing diets showed normal body weight development and behaviour without any signs of adverse effects. Furthermore, no macroscopic lesions or histopathological changes were identified in the pancreas.

We conclude that the changes in pancreas weights in male animals, which were only significant in trial A and not dose dependent, are not toxicologically relevant and not related to the feeding of the GMO-containing diets. If one takes into account that also the increase in glucose levels in male animals in trial A was not dose related and that there were no differences at all in the glucose levels between the different groups in trial B, the claimed link to an effect on the pancreas is unjustified.

As mentioned by Zeljenková et al. (2014) in the Discussion section, a 1-year feeding study with the MON810-maize is being performed at the present time by the GRACE consortium. Based on the results of the ongoing 1-year trial with maize MON810, the relevance of the findings in the 90-day feeding trials will again be assessed.
